# Information and support for cancer patients and their relatives - evaluation of central contact point enquiries: a retrospective analysis

**DOI:** 10.1186/s12913-025-13342-z

**Published:** 2025-09-08

**Authors:** Theres Fey, Julia Kasprzak, Julia Ernst, Franziska Weiher, Marina Schmid, Nicole Erickson, Brigitte Kühnel, Wuthichai Prompinit, Sylvia Tanzer-Küntzer, Hana Algül, Volker Heinemann, Daniel Nasseh

**Affiliations:** 1 Comprehensive Cancer Center (CCC Munich LMU), LMU Hospital, München, Germany; 2 Bavarian Cancer Research Center (BZKF), LMU Hospital, München, Germany; 3 Comprehensive Cancer Center Munich (CCC Munich), LMU Hospital and TUM Hospital Klinikum rechts der Isar, München, Germany; 4 Comprehensive Cancer Center (CCC Munich TUM), TUM Hospital Klinikum rechts der Isar, München, Germany; 5 Department of Internal Medicine II, TUM Hospital Klinikum rechts der Isar, München, Germany

**Keywords:** Cancer information, Patient referral, Second opinion, Consultation, Supportive care, Information needs

## Abstract

**Background:**

The Comprehensive Cancer Center Munich has established a central contact point for cancer patients and their caregivers, which is associated with a multidisciplinary supportive care center. The platform facilitates multifaceted enquiries about access to supportive care, second opinions and specialist care. The aim of this study was to investigate the utilization of the contact platform during a period of 31 months.

**Methods:**

The analysis was sourced from data regarding contact information gathered between January 1, 2022, and July 31, 2024. The dataset was completed by Privacy Preserving Record Linkage, using the tumor documentation data.

**Results:**

Within 31 months, a total of 1226 enquiries were documented, originating from 1058 distinct individuals. The three most common requests were for second opinions (39%), supportive care consultations (37%) and treatment options (16%). 51.5% of the individuals of the cohort were younger than 60 years old. Age groups ≥ 70 years and above were underrepresented compared to the general population of cancer patients in Germany. Women were overrepresented (65.1%). Supportive counseling was mostly requested by women (75.5%). 72.5% of the individuals that contacted the service were patients and 27.5% relatives or friends. While enquiries about second opinions and supportive care mostly came directly from the patients, the majority (55%) enquiries about treatment options were made by associated persons.

**Conclusions:**

This dataset provided valuable insights as to how the central contact platform service is utilized. These insights will serve to properly address patients’ information needs thereby improving access for underrepresented groups.

**Supplementary Information:**

The online version contains supplementary material available at 10.1186/s12913-025-13342-z.

## Background

Around 500,000 people in Germany are diagnosed with cancer every year [1]. At the same time, diagnostic and treatment methods are rapidly evolving and becoming more complex [2, 3]. There were around 4.65 million cancer patients and cancer survivors in Germany who have or had cancer in 2017 [4]. Given the rising number of people affected by cancer, it is essential to provide a comprehensive spectrum of care that encompasses both therapeutic and supportive services from the point of diagnosis and throughout the trajectory of care.

In today’s understanding of patient care, the patient is at the center of medical care. The aim of patient-centered care is to provide patients with treatment, care, and information that is tailored to their individual needs. The need for patient information and accompanying services for cancer patients, such as access to second opinions and support care, as well as information regarding study participation is growing [5–7]. However, it is becoming increasingly complex for patients to both locate these services and, particularly in the age of the internet, ascertain their credibility. Even when a suitable service provider has been identified, it is often challenging for patients to find the appropriate contact point. All these hurdles can lead to frustration for the patient and may result in the reluctance to accept assistance even if they would benefit the patient.

To address this issue, the Comprehensive Cancer Center Munich (CCCM) has established a central contact point to assist in facilitating enquiries about access to supportive care services, second opinions (i.e., independent expert assessments of an existing diagnosis or treatment plan) and specialist care, as well as providing guidance and recommendations for participation in clinical studies. The CCCM is a collaboration between the cancer centers of both Munich University Hospitals, namely the hospitals of the Ludwig-Maximilians-University (LMU) and the Technical University Munich (TUM), which, in turn, represent and support the various certified organ cancer centers at the respective sites.

While central contact platforms at CCCs or other large cancer centers are not uncommon and well established in Germany [8–10], the Munich site has a unique feature: the CCC patient hotline. This hotline is affiliated with a multidisciplinary supportive care center called the “Patientenhaus” (Supportive Care Center) [11]. The Patientenhaus bundles various counseling services from professionals employed with the Bavarian Cancer Society, a non-profit organization called “lebensmut e.V.”, and the CCC at one site. Services provided include: psycho-social support services, consultations with qualified specialists for nutrition and complementary medicine, a referral to second opinions, and information regarding participation in clinical studies. All enquires are coordinated centrally as part of the central contact platform.

The aim of this study was to gain insights into information needs of cancer patients and to assess how the use of the contact platform has developed over a period of 31 months (2 years, 7 months). The patient cohort was to be analyzed according to in terms of gender and age distribution, diagnosis, and reason for the request. To this end, the enquiries at the central contact point that were made between January 2022 to July 2024) were categorized and retrospectively analyzed. In order to identify which of the patients contacted the service and subsequently received treatment at the CCC Munich, we employed a privacy-preserving record linkage method [12] to integrate information from the internal tumor documentation data [13, 14] ensuring data privacy throughout the process.

## Methods

The methodological process encompasses data acquisition, data preprocessing, record linkage, and statistical analysis. Supplementary Fig. 1 provides an overview of the entire process in this retrospective study, with detailed descriptions of each step outlined subsequently.

### Data acquisition

The central contact platform is operated by CCC Munich as a low-threshold coordination service and complements existing local contact points at organ-specific cancer centers. Patients and caregivers may still contact these centers independently. A team of trained staff members at the central office receives all enquiries and manually triages them based on the request - either by responding directly or forwarding to the appropriate local service. Triage is based on internal directories and established communication paths, allowing for flexible and individualized handling of requests. The team regularly participates in weekly scheduled coordination meetings, ensuring up-to-date knowledge of available services and maintaining direct communication with all sub-centers. During patient or caregiver interactions, a standardized set of basic information (e.g., role of the enquirer, type of request) is routinely documented for service purposes, independent of this study.

The dataset utilized in this retrospective study was sourced from contact information gathered on weekdays between January 1, 2022, and July 31, 2024, through telephone enquiries, and to a lesser extent, through e-mail enquiries directed to the central office of the CCC Munich. As of June 2022, this service additionally facilitated referrals to the services provided by our supportive care center. Typically, provision of names and occasionally birth dates or an often-generalized diagnosis of cancer, along with contact information such as email addresses or phone numbers, and an indication of whether the individual is a patient or an associated person (relative, friend or another caregiver) was provided. Additionally, it was documented to whom the information was forwarded alongside some individual comments. This data was compiled into an Excel spreadsheet and checked for quality assurance. However, in order to keep the service accessible, data collection during enquiries was intentionally non-intrusive; missing information (e.g., diagnosis, age) was accepted without follow-up questions. The quality of the data was further compromised due to the partially telephonic and unstructured nature of the data collection process.

### Data preprocessing

Due to privacy laws, the team responsible for direct patient contact undertook the task of data preprocessing. which included reviewing the records and manually filling in missing values (e.g., gender or diagnosis) based on available information. Initially, first names and last names were separated, and additional comments such as Mr., Mrs., or Dr. titles were removed. As it was not uncommon for individuals to call multiple times, a unique individual ID was subsequently assigned, and based on the available contact information, this ID could be associated with multiple entries, ensuring that patients were counted only once in the statistical analysis. Gender was identified based on the first name for a significant portion of cases. In cases of ambiguity, gender was left blank. Individual cancer diagnoses recorded in free text, which were often provided during the conversation, were organized into generalized categories. The categories were (with slight deviations) based on certifiable (organ) entities defined by the German Cancer Society [15]. Furthermore, classification was conducted regarding the preferred method of patient re-contact. For instance, providing an email address was evaluated as a digital preference. Finally, the approximate geographic locations, indicated by the first two digits of the postal codes, were derived from available telephone numbers (e.g., 089 corresponds to Munich).

To assess the extent of utilization of support services, participant lists of support services located directly within the CCC Munich were cross-referenced with the analysis data set. If an individual utilized any of these support services, this was noted. The cross-referencing was conducted directly by the team members of the supportive care center, who were granted restricted access to the caller information of individuals who had communicated with the central contact point regarding a consultation request. This process was carried out in accordance with an explicit confidentiality agreement.

### Privacy preserving record linkage

To determine whether individuals who contacted the central platform - e.g., to request a second opinion or supportive care - subsequently received treatment or utilized services at one of the two associated university hospitals, we performed a deterministic Privacy Preserving Record Linkage [12] with the respective tumor documentation data. The procedure employed for this purpose was as follows: Individual ID, first name, last name, date of birth, and generalized ICD-10 group were transcribed to a separate file. Employing a Python script [16], first names and last names were standardized following the UNICON [17] rule set. Anticipating variations in names received over the phone, such as “Marc” or “Mark,” the codes underwent additional processing using the Cologne Phonetic algorithm [18], which aligns such variants (specific for the German language) to the same code. The different applied standardization rules are listed in Supplementary Table 1.

It is noteworthy that the final standardization step involves a one-way encryption using a hashing algorithm (from the SHA-3 family) [19, 20], which has been supplemented with a “secret salt” which is a random string of text added to the original text [21]. This ensures protection against dictionary attacks [22], and the name attributes cannot be re-identified without the original list. Hashing was also applied to the date of birth.

The newly generated file was dispatched to the site-specific IT staff (associated with the CCCM) responsible for managing the tumor documentation systems at each of the two associated university hospitals. A standardization script similar to the one used previously and incorporating the same salt, was supplied to the same IT staff, who subsequently applied it to minimal exports of the tumor documentation data. Once both datasets were standardized, they were prepared for matching.

Typically, a deterministic record linkage, based on matching first name, last name, and date of birth, achieves a specificity of over 99%, but with varying sensitivity [23]. We chose the diagnosis category as the fourth matching variable due to frequent absence of the date of birth and related problems with data sensitivity. Matches were considered for cases that agreed in at least three of the matching variables between the analysis data set and the tumor documentation data. While this approach may lead to a reduction in specificity, it is anticipated to significantly improve sensitivity. While the exact increase in false positives is not determinable, we expected it to remain statistically acceptable.

Upon identifying a match, the list of four matching variables, which additionally included the previously assigned individual ID, was supplemented with the year of birth stored in the tumor documentation system. Additionally, the disease entity (grouped in the same manner), as well as the first (hospital internal) date of treatment and the first two digits of the postal code where the patient resides were appended.

The matches, along with the additional details, were subsequently transmitted back (using the LMU-Databox) to the data management team of the supportive care center (Patientenhaus). This additional information was then integrated into the analysis data set using the individual ID as the reference. This procedure facilitated improved accuracy of birth dates and disease profiles. Moreover, it enabled the verification of whether patients, subsequent to the initial enquiry regarding a second opinion, had attended one of the clinics and subsequently undergone treatment.

### Statistical analysis

The extended dataset underwent formal anonymization, involving the removal of identifying information. In this process, the ‘year of birth’ field was substituted with the age at contact, calculated using the ‘year of birth’ and the ‘date of contact’ field. Furthermore, patients were flagged if they received treatment at LMU or TUM Hospital based on merged date fields from the tumor documentation system, with a distinction whether these treatments occurred after or before the initial contact. Subsequently, the anonymized dataset was transferred to the data science team of the CCCM. The ensuing data analysis was also conducted using Python version 3.8.8 [16].

## Results

### Overview of enquiries

**Fig. 1:**
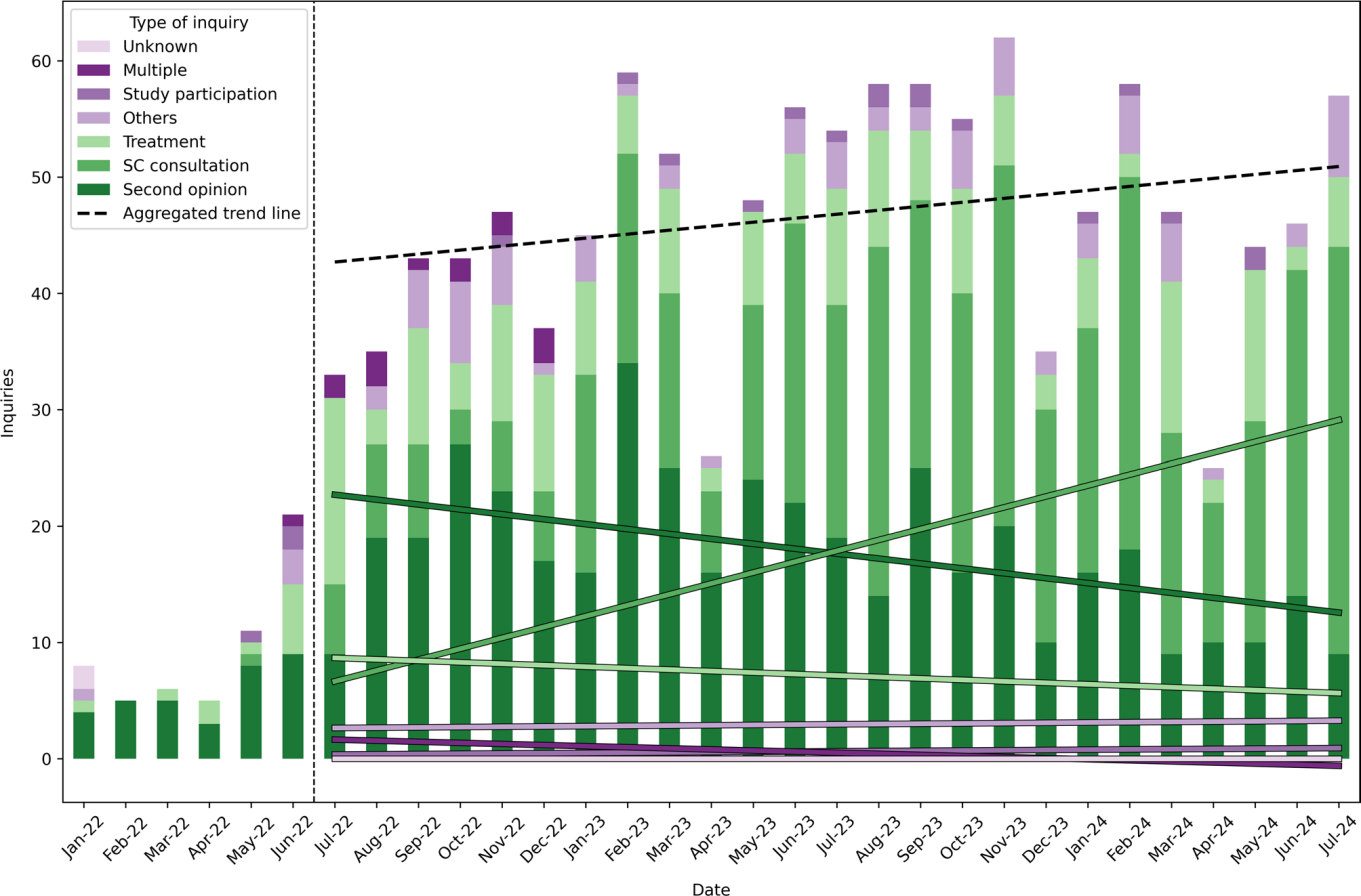
This graph depicts the number of enquiries received at our facility over the 31-month period. Regression lines indicate trends for the different types of enquiries. Since the data collection was incomplete until June 2022, regression lines were only calculated since July 2022. SC consultation (supportive care consultation) comprises offers including psychosocial support, as well as consultations with specialists from the areas of nutrition and complementary medicine.

Throughout the designated timeframe (31 months), a total of 1226 enquiries were documented, originating from 1058 distinct individuals. Notably, 911 (86%) individuals contacted central office once, 147 (14%) individuals engaged in multiple subsequent interactions. Figure 1 illustrates the temporal distribution of enquiries based on the month of their occurrence and the reason for the request. As the systematic documentation of the queries began in July 2022, the regression lines were only generated from this point onwards.

From July 2022 to July 2024, an increase in requests for supportive care consultations could be observed (Fig. 1). The green regression line demonstrates a tripling of these requests in this time frame. In contrast, monthly enquiries about second opinions decreased within the same period (Fig. 1, blue line). Therapy requests decreased slightly (orange line), whereas enquiries on other topics such as study participation, other reasons, or multiple requests remained constant over this period. The overall trend line shows a slight increase in enquiries. Requests dropped in April and December in both 2023 and 2024.

Given that a substantial portion of enquiries were received by telephone, notable limitations in data quality were expected. While the overall trends remain robust, several key data fields - such as diagnosis or date of birth - were frequently incomplete or inconsistent. To complement the enquiry dataset with verified clinical information, we conducted a Privacy-Preserving Record Linkage with the tumor documentation systems of two university hospitals. In total, 148 validated matches were used to append previously unavailable clinical data - such as confirmed diagnoses and treatment initiation dates - and to correct or validate selected entries in the original dataset. An overview of data completeness is provided in Supplementary Tables 2, and further details on the linkage process can be found in Supplementary Tables 3 and Supplementary Note 1.

### Demographics

**Fig. 2:**
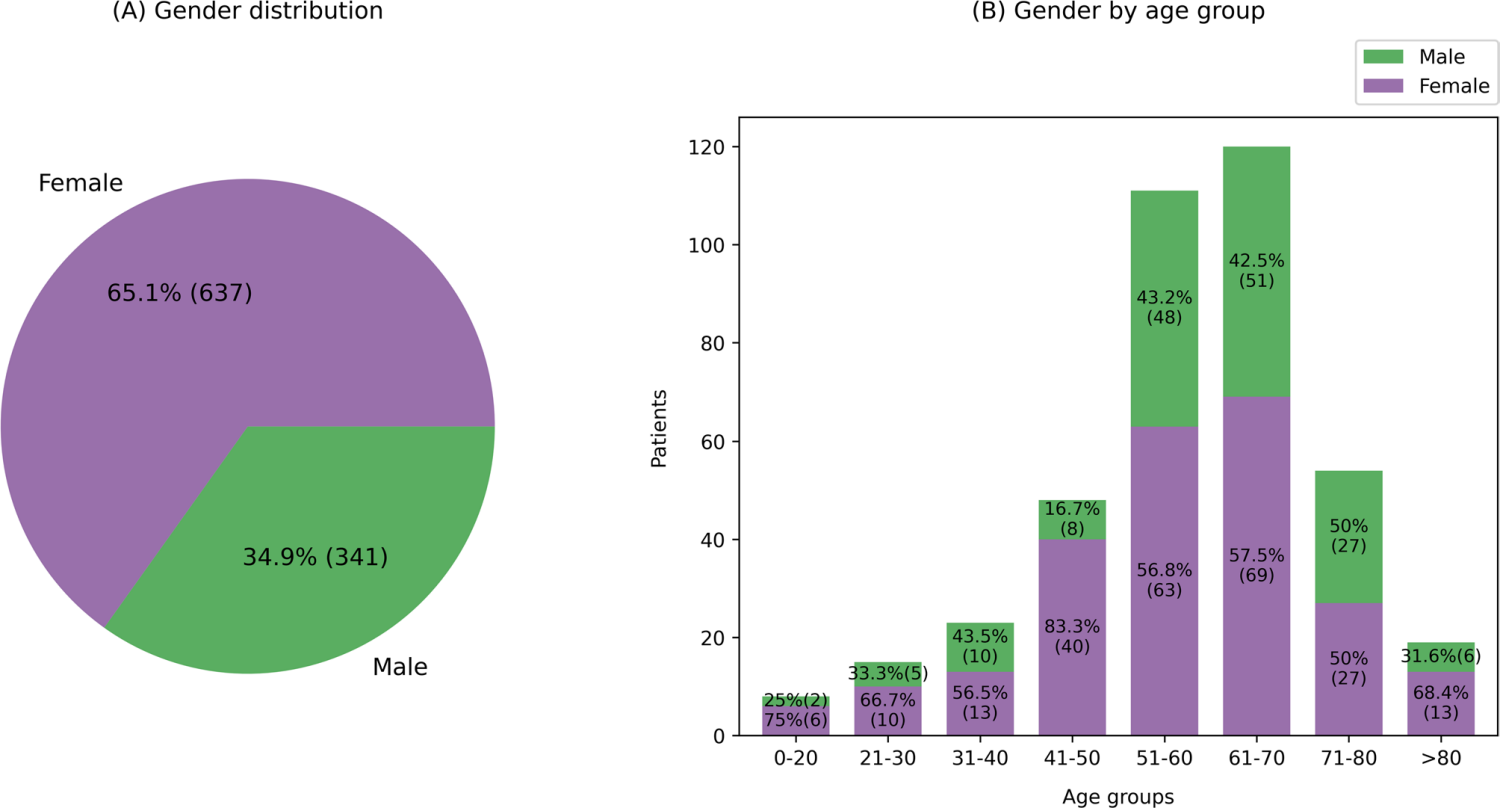
**A** Gender distribution. **B** Gender by age groups of enquiring individuals. Individuals with multiple enquiries are counted multiple times. Missing Data: 6.9% of gender and 59.0% of age groups are unknown (see Supplementary Table 2) and therefore were not included in this graph.

65% of all enquirers were women. Hence, approximately twice as many enquiries were initiated by women (women: 65%, vs. men: 35%) (Fig. 2A). Since the epidemiologic age distribution of the cancer cohort in Germany is 47% women and 53% men [24], men are clearly underrepresented. Over 50% of the cohort was younger than 60. Patients aged 70–80, but even more drastically, 80 and above are not as prevalent in our cohort compared to the actual epidemiology [24–26] (Fig. 2B).

**Fig. 3:**
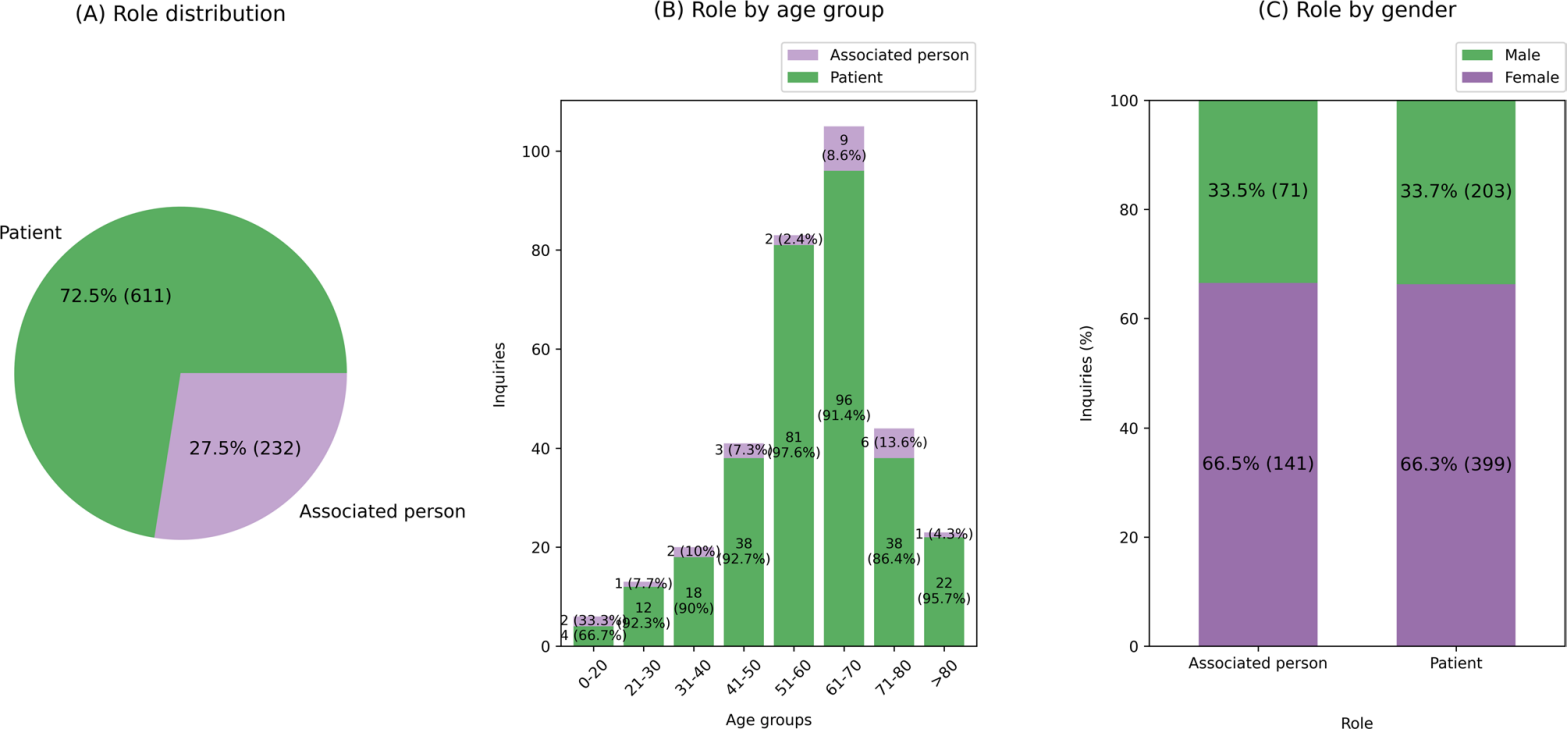
**A** Role distribution, **B** role of enquiring individual per age group of patients, **C** role by gender of enquiring Individual. Individuals with multiple enquiries were counted multiple times. Note: 31.2% of roles and 59.0% of age groups are unknown (see Supplementary Table 2) and therefore were not included in this graph.

72.5% of the people were patients and 27.5% were associated persons (Fig. 3A). In the age group 0–20 years, the proportion of enquiries from associated persons is higher than in the other age groups (Fig. 3B). In both the patient and the associated person roles more enquiries were made by women than by men (Fig. 3C, patient role: 66% women, associated person: 67% women).

### Type of enquiries by gender and role

**Fig. 4:**
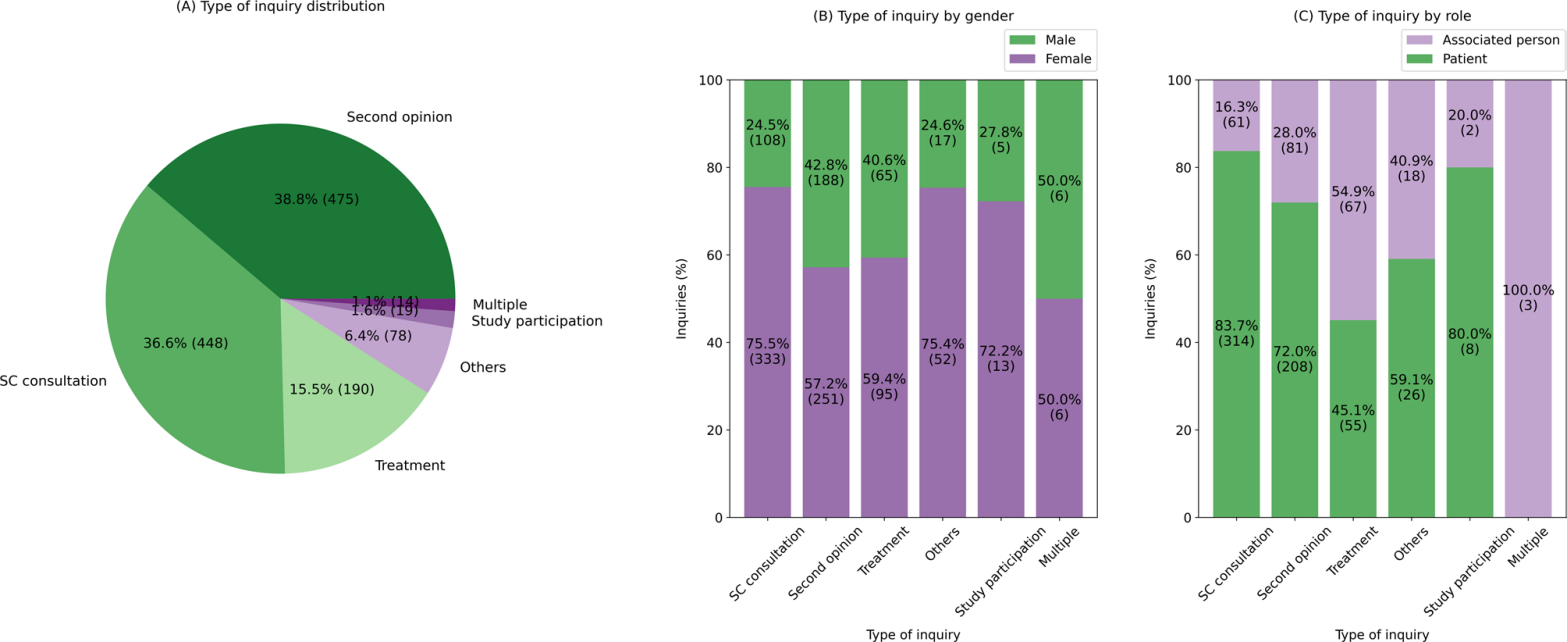
**A** Type of enquiry distribution, **B** Type of enquiry by gender, **C** type of enquiry by role of enquiring individual. Individuals with multiple enquiries are counted multiple times. Note: 0.2% of type of enquiries are unknown (see Supplementary Table 2) and therefore were not included in this graph

Figure 4A shows the proportion of enquiries on the specific topics. The three most common requests were for second opinions (39%), supportive care consultations (37%) and treatment options (16%). Enquiries on multiple topics were only made in 1% of cases.

The three most frequent enquiry types were second opinions (39%), supportive care (37%), and treatment requests (16%). While second opinions and treatment requests were requested by 57% and 59% women, respectively, the proportion of requests for supportive counseling by women was almost 76% (Fig. 4B). The majority of second opinion and consultation requests were made by the patients themselves, whereas 55% of treatment requests came from associated persons (Fig. 4C).

**Fig. 5:**
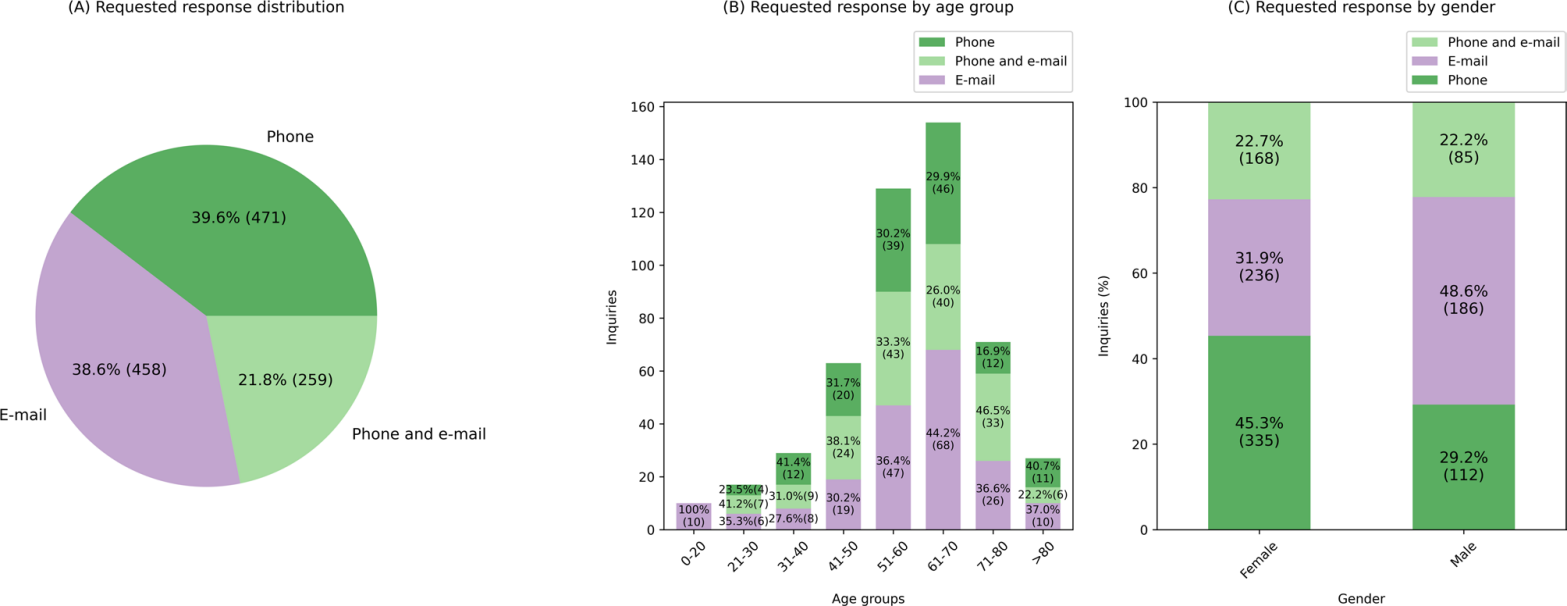
**A** Requested response distribution, **B** by age group, **C** by gender of enquiring individual. Individuals with multiple enquiries are counted multiple times. Note: 3.1% of requested responses are unknown (see Supplementary Table 2) and therefore were not included in this graph.

Additionally, the contact preferences were analyzed. There was a choice of receiving responses either by e-mail (written contact) or telephone (verbal contact), or both (Fig. 5). Overall, there was no clear preference among enquirers regarding the use of phone or email (Fig. 5A). However, the 0–20 age group clearly preferred feedback by e-mail. For all other age groups, all 3 options phone, e-mail and phone + e-mail were represented (Fig. 5B). The option via e-mail (written contact only) was preferred by men (49%), whereas women had a slight preference for being contacted by phone (verbal contact only) (45%) (Fig. 5C).

Comparable to the nationwide incidence rates, female enquirers were most frequently affected by breast cancer (35%) and males by prostate cancer (11%) [24] (see Supplementary Fig. 2 for a full overview of tumor site distribution). Since a larger percentage of enquiries came from women, tumors commonly associated with female are overrepresented in the types of cancer of the enquirers (44% breast cancer and gynecological tumors).

Supplementary Fig. 3 provides an overview of the types of cancers associated with the enquiries. Additionally, it indicates whether the information originates from the original or supplementary tumor documentation. As a variation of stratification, supplementary Fig. 3 describes the distribution of general cancer groups by role.

### Conversion into actual service use

**Fig. 6:**
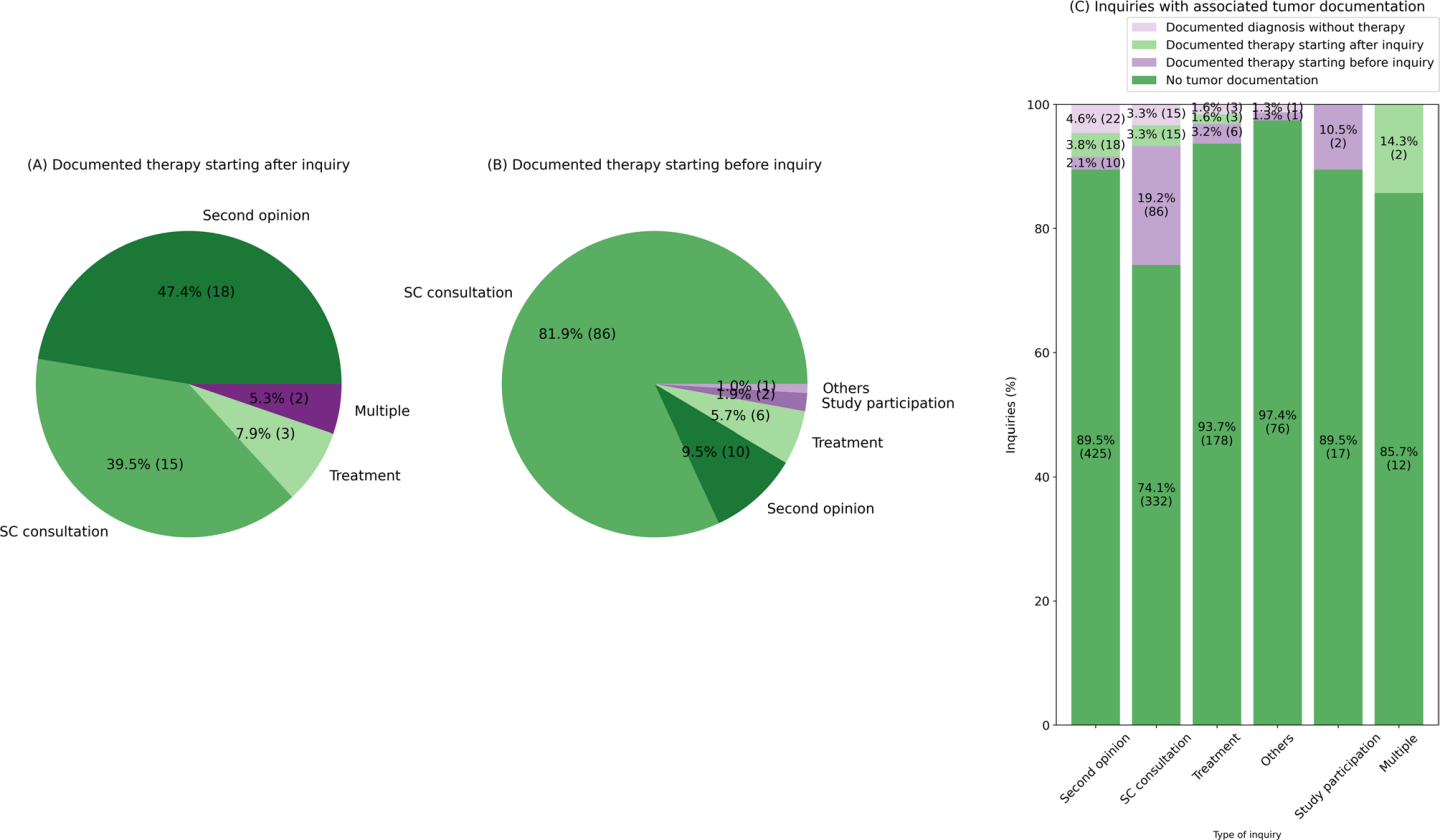
Type of enquiry associated with local treatment at one of the two university hospitals (case documented in the central tumor documentation). A Proportion of type of enquiry of patients for which a documented therapy after the enquiry was observable. B Proportion of enquiries already having associated tumor documentation data before the enquiry as well as enquiries occurring prior to local treatment. C Proportion of enquiries per type of enquiry with associated tumor documentationType of enquiry associated with local treatment at one of the two university hospitals (case documented in the central tumor documentation). A Proportion of type of enquiry of patients for which a documented therapy after the enquiry was observable. B Proportion of enquiries already having associated tumor documentation data before the enquiry as well as enquiries occurring prior to local treatment. C Proportion of enquiries per type of enquiry with associated tumor documentation.

Furthermore, it was examined whether individuals who contacted the central platform had been treated at either one of the two university hospitals associated with CCC Munich before or after their enquiry (Fig. 6). This was verified through the linkage of central tumor documentation data.

47% of the patients who had already been treated at one of the two hospitals before contacting the central contact platform sought a second opinion, while 40% requested a supportive care consultation (Fig. 6A). The majority of patients who were treated at one of the hospitals after their enquiry requested a supportive care consultation (82%) (Fig. 6B). 3.8% of patients who requested a second opinion were subsequently treated in one of the two hospitals (Fig. 6C). Of the patients who requested a supportive consultation, 19.2% were already patients at one of the two university hospitals.

We also evaluated how many of the individuals who requested supportive counseling actually received counseling at the supportive care center. 78% of these requesters received supportive counseling (see Supplementary Fig. 4 A). There were no age-specific anomalies (Supplementary Fig. 4B) or gender-specific differences in this context (Supplementary Fig. 4 C).

### Regional distribution

The geographic origin of the enquiries was available for 376 out of 1226 enquiries (31%) within Germany, (see distribution of enquiry map in Supplementary Fig. 5). Additionally, 124 international enquiries were recorded, based on further information provided by the caller. In total, 363 enquiries originated from the state of Bavaria (96.5%), The majority of the 376 enquiries originated from around the Munich area (255, 68% - Munich and Munich region). 108 (29%) originated from the rest of Bavaria. 7 enquires stemmed from Baden Württemberg (2%) and 6 from the rest of Germany (1.5%). 79% of the requests for supportive care counseling came from the Munich region. 19% of these enquiries came from the rest of Bavaria. 62% of treatment requests came from the Munich region and 38% from the rest of Bavaria. 49% of requests for second opinions came from Munich and the surrounding area. 46% of these requests came from the rest of Bavaria (see Supplementary Table 4 for information on whether enquiries originated from the immediate vicinity or from more distant areas).

## Discussion

The contact platform serves as a low-threshold gateway to information and services. In total, it was contacted over 1,200 times during the period of 31 months that was examined, corresponding to a monthly frequency of 40 contacts. The three most common requests were for second opinion (39%), supportive care consultations (37%) and treatment options (16%). Only 1% of the individuals contacting the service had requests on multiple topics, indicating that patients mainly used the platform for specific requests. The drop of enquiries in April in 2023 and 2024 can possibly be explained by the fact that a patient event organized by the CCC Munich that focuses on supportive care, takes place at the end of April. This may have resulted in patients requesting the information needed directly at the event and not via the contact platform. Furthermore, since the staff of the contact platform is engaged in preparing the event, this may have caused a delay in the response time to enquiries at the contact platform in this month. The drop in the December months can be explained by the Christmas holidays.

The establishment of the CCC Munich supportive care center coupled with its continuous expansion resulted in a clear increased demand for supportive care consultations. This was in part achieved through extensive outreach activities, such as promotion of the offerings at associated hospitals and clinics, as well as an array of press activities. The data also indicated that the demand has not yet been met and that there is further potential for growth. Whereas the majority of requests for supportive care counseling came from the Munich region, enquiries regarding second opinions were requested to a higher extent from the rest of Bavaria. The overall demand for second opinions slightly decreased during the time period used as a basis for this analysis. However, compared to the reports from other literature the total number of enquiries about second opinions we received was similar to other centers. Ruetters et al. showed that between 6.5 and 36% of patients search for a second opinion [27]. In 2020 Olver et al. found that 16.1% of patients in Australia had sought a second opinion about their cancer treatment [28]. As it is important that patients have access to independent opinions in order to ensure that they receive the best possible care, it has been a consensus in Germany that cancer patients should have access to a free second opinion. In fact, Lehmann et al. showed that second opinions can yield psychological benefits by reducing patients’ uncertainty, but the added medical value remains debatable [29]. However, second opinions require additional resources and time, which can be challenging in today’s healthcare system. Furthermore, the costs are only partially reimbursed. To systematically introduce second opinion in oncology, a structured program considering costs and medical advantages therefore seems essential.

In terms of the demographics, we observed that women were overrepresented (65.1%). These findings were similar to those of Squiers et al. and Reifegerste et al. [10, 30] which showed that the majority of calls to the US and the German Cancer Information Service were made by female (68.1% and 65.5%) patients. In this study, a comparable distribution in gender was found in both groups of the enquiring cohort – patients and their caregivers. For this reason, cancers that affect women were also overrepresented in the analysis. Supportive counseling in particular was largely requested by women (female 75.5% vs. male 24.5%). The fact that men are less likely to make use of support services is also reflected in the current literature [31, 32]. This is especially relevant in outpatient care, where patients often do not receive proactive support. However, we believe that we could also tailor the offerings more towards men. For example, Wünsch et al. proposed 4 actions: [1] Provision of evidence-based information which actively recommends supportive care specifically to men [2], use men instead of women in publicity campaigns (e.g., in flyers, videos) [3], implement structural changes in the counseling services (e.g., introducing evening appointments) [4], initiate activities that bring men together (e.g., sailing) [33, 34].

While 51.5% of the individuals of the cohort were younger than 60, individuals aged 70 and above were underrepresented compared to the general population of cancer patients in Germany. A similar distribution was found by Squiers et al. [30] in an analysis of callers of the US cancer information system (age < 60, 51,2%). This may be caused by technical, physical, and digital barriers. It has been shown that older people are less likely to use the internet, resulting in reduced access to online information. Since information regarding the CCC contact platform is mainly communicated digitally, this may result in the older patient cohort being less aware of it. In addition, older cancer patients may be less mobile or suffer from geriatric symptoms [35]. Therefore, in order to support patients over the age of 65, a program was developed at the CCC supportive care center that specifically addresses the needs of older patients. In addition, it seems important that this demographic has access to printed information instead of digital information. This can be achieved by providing patients with information folders with printed information. Since our data indicated that associated persons like relatives, friends and other caregivers among the older patients were not requesting the contact platform as frequently as compared to associated persons among the younger patient collective, an increasing involvement of associated persons may also help to improve information on supporting offers.

Looking at the overall cohort, there was no clear preference for verbal or written contact. However, men tended to prefer communicated through email, while women prioritized verbal contact per telephone, which confirms Coffman’s observation that women are more vocal than men [36]. Although gender specific communication styles exist [37, 38], it is important to recognize that gender is one of many factors (e.g. age, education, ethnicity) that can influence communication behavior [39].

About 28% of those who contacted the platform were associated persons (family members, friends and other caregivers). This finding is comparable to that of Reifegerste et al. which showed that 35% of callers contacting the German Cancer Information Service were family members or friends [10]. While enquiries about second opinions and supportive advice were mostly initiated by the patients themselves, the majority (55%) of treatment enquiries were made by associated persons. This may be due to the fact that at the time of diagnosis, when suitable treatment options need to be found, patients may not be in good health or may be mentally overwhelmed by the new situation and need their relatives’ help to find a specialized clinic. Interestingly, only 2% of patients who requested treatment were subsequently treated at one of the two hospitals. For those seeking a second opinion, 4% were ultimately treated at one of these hospitals. However, it is important to note, that the record linkage connecting the data with data of the tumor documentation of the hospitals was conducted with incomplete and imprecise data, leading to some individuals not being recognized as patients. Consequently, the actual Figures are likely to be somewhat higher. Additionally, it should be emphasized that the majority of treatment requests are made directly to the clinics or participating organ cancer centers, bypassing the central contact platform. Notably, 20% of patients who requested supportive counseling were already undergoing treatment at one of the two clinics. This indicates that the services offered by the supportive care center are well known by the health care professionals and the patients of both clinics. This is achieved through extensive advertising of the center in the form of flyers and patient information folders. Once again, due to the quality of the data used in the record linkage process, the actual numbers are likely to be somewhat higher.

This study provides an insight into the use of a central contact platform of a German Comprehensive Cancer Center. Two comparable studies which examined the information needs of US cancer patients who contacted the National Cancer Institute’s (NCI’s) Cancer Information Service [30] and of callers of a large German cancer information service [10] were published in 2005 and 2021. Patient information contact services have been established at Comprehensive Cancer Centers throughout Germany and are also provided by the German Cancer Information Service [8] and the Public Citizen Hotline of the Bavarian Center for Cancer Research [9]. The platform of the CCC Munich has a unique feature, since it also coordinates the services provided by the recently established supportive care center. This aligns well with major German initiatives such as ONCONNECT or the National Decade Against Cancer, which aim to empower strong centralized centers to extend their expertise outward, thereby enhancing outreach and enabling patients in peripheral regions to benefit [40, 41]. The central contact point and the supportive care center are located in close proximity and are coordinated by the same office. As a consequence of this harmonization of services, patients and their relatives can easily gain access to a wide range of information and services via one single contact. This saves patients’ time as they no longer need to seek various separate service points out and therefore can avoid numerous, and partly unnecessary contact enquiries. Moreover, well-coordinated communication structures enable rapid referrals. The service therefore can be highly beneficial for patients and their relatives, improving overall patient information and patient care. Hence, the present data can be used to more effectively address the full scope of cancer patients’ information needs.

### Limitations

The central contact platform serves as a gateway to information and services. Initially, only the data necessary for addressing and forwarding the concerns was documented, with additional information being recorded only if provided by the enquiring individual. Consequently, the data collected for the present evaluation was sometimes incomplete or prone to errors in certain fields. This reflects our low-threshold design: enquiries were accepted even when key information was missing or only partially available, in order to ensure inclusivity and ease of access for patients and caregivers. While surnames and contact information were generally available, information such as first names, birth dates, and details on the type of illness or tumor were occasionally incomplete. While the use of more advanced IT systems could potentially reduce the extent of missing data, we believe this would conflict with the core purpose of the contact platform. The primary objective is not data collection, but to support patients and their relatives in times of crisis. Especially in the context of severe illnesses such as cancer - and particularly in an era increasingly shaped by artificial intelligence - we consider direct, human contact to be essential. Empathy and emotional sensitivity remain uniquely human traits, and we believe that patients seek and value this type of connection when navigating their illness.

Thus, all data, especially the data supplemented through record linkage, must be interpreted with caution. Although the data is reasonably close to reality and sufficient for identifying trends, it lacks complete precision. It should also be noted that, although the study covers a period of 31 months, it includes only approximately 1,200 contact enquiries. While this is a substantial number, an even higher volume of enquiries would be desirable to ensure stronger generalizability of the findings. It is also important to note that this study did not involve a prospective data collection; rather, it relied on a retrospective analysis of existing routine data. No additional burden was placed on patients or callers, and the data was handled with care in accordance with Article 27 of the Bavarian Hospital Act - ensuring that no individually identifiable information left the site. In that regard, the project underwent a data protection review at both university hospitals and was deemed acceptable by the Ethics Committee of the LMU. It was approved under application number 24–0871.

## Conclusion

This study provides important insights into how a central contact platform can support cancer patients and caregivers in navigating cancer care. Over a 31-month period, the service received 1,226 enquiries, most frequently related to second opinions (39%) and supportive care (37%). The majority of enquiries were made by women (65%), while older patients (≥ 70 years) were less frequently represented compared to national cancer incidence data. This indicates potential access barriers for specific subgroups. At the same time, the platform proved effective in facilitating access: for example, 78% of individuals who requested supportive counseling went on to receive services. In summary, the central contact point offers low-threshold access to a wide range of services. Patients can get access to a wide range of information, treatment opportunities, second opinions, and supportive care via one single contact. The adaptability of this concept makes it particularly promising for other locations, where diverse patient populations may face unique challenges in accessing similar coordinated care efforts. Establishing similar central contact points could therefore facilitate the access to high-quality, integrated care that meets individual needs, fostering greater inclusivity, and providing comprehensive support for a broader population.

## Supplementary Information

Supplementary Material 1.

## Data Availability

The datasets used and/or analysed during the current study are available from the corresponding author on reasonable request.

## Abbreviations

CCCM: Comprehensive Cancer Center Munich
NCI: National Cancer Institute
