# Supplementary material for: Information and support for cancer patients and their relatives - evaluation of central contact point enquiries: a retrospective analysis
Source: BMC Health Serv Res. 2025 Sep 8;25:1192. doi: 10.1186/s12913-025-13342-z (PMC12418670; doi:10.1186/s12913-025-13342-z)
Supplement: Supplementary file 1 — Supplementary Material 1. [file 12913_2025_13342_MOESM1_ESM.pdf]

SUPPLEMENTARY MATERIAL

Supplementary Figure 1: *Once an enquiry is initiated (1), the individual's data is compiled in a contact sheet (2). This information is then used by the central office to establish contact (3) with either hospital staff or personnel from the supportive care center (4). Direct engagement between the individual and these services or hospital staff may follow (4), potentially leading to program enrollment or hospital admission (5). In the latter case, admission details are documented in the CCCM's tumor documentation system. For this retrospective analysis, the contact data undergoes cleaning and preprocessing (6), with name-related fields stored as free text under the designation "IDAT." A set of matching variables is then standardized and partially hashed (#IDAT), and the resulting data is exported to a separate file (7). This file is securely transmitted to the CCCM's IT staff (8). Simultaneously, tumor documentation data is standardized and hashed using the same algorithms employed for creating the Analysis Matching Data (9). Following this, both datasets undergo a privacy-preserving record linkage process (10). Linked data from both hospitals, along with participant lists from services of the supportive care center, are finally merged, allowing for the complete removal of IDAT and rendering the dataset ready for statistical analysis (11).*

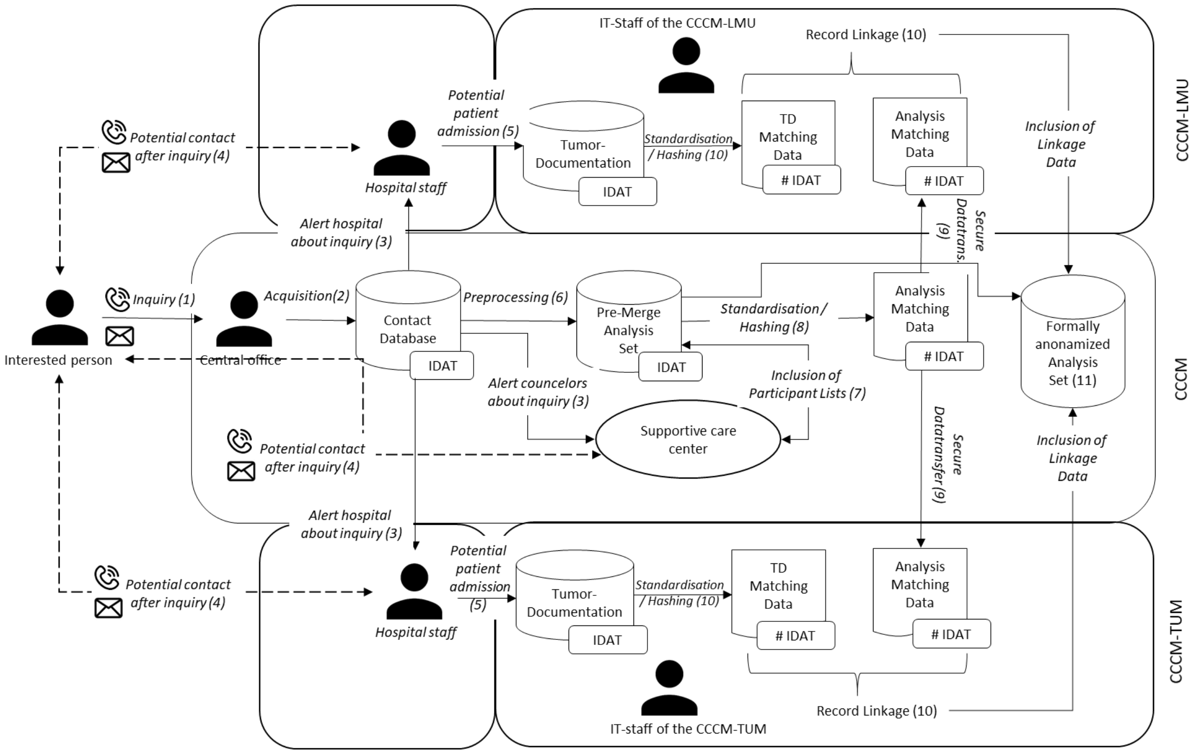

Supplementary Table 1: *Standardization rules applied on the name fields for the core dataset as well as the tumor documentation data sets.*

**Method**	**Before Standardization (Example)**	**After Standardization (Example)**
Convert special character	von LópeZ-Müller	von LopeZ-Müller
Remove Name additions	von LopeZ-Müller	LopeZ-Müller
Convert German umlaut	LopeZ-Müller	LopeZ-Mueller
Remove non-alphabet symbols	LopeZ-Mueller	LopeZ Mueller
Only keep first name	LopeZ Mueller	LopeZ
Transform to upper case	LopeZ	LOPEZ
Trim potential whitespaces	´ LOPEZ `	´LOPEZ`
Apply cologne phonetic	LOPEZ (same for e.g. LOPES)	50108
Hashing	50108	Hashcode, e.g.: kFZKQdw+7G

Supplementary Table 2: *The table provides information on the completeness of the recorded data. Additionally, it indicates the extent to which the data could be supplemented based on record linkage with tumor documentation data, as well as the extent to which discrepancies between the data were corrected using the tumor documentation data.*

	**Available**	**Missing**	**Suppl. TD information**	**Correcting TD information**
**First name**	988 (80.6%)	238 (19.4%)	0 (0%)	0 (0%)
**Last name**	1151 (93.9%)	75 (6.1%)	0 (0%)	0 (0%)
**Date of birth**	400 (32.6%)	723 (59%)	103 (8.4%)	0 (0%)
**Gender**	1138 (92.8%)	85 (6.9%)	3 (0.2%)	3 (0.2%)
**Role**	843 (68.8%)	383 (31.2%)	0 (0%)	0 (0%)
**Diagnosis**	1073 (87.5%)	150 (12.2%)	3 (0.2%)	18 (1.5%)
**Type of enquiry**	1224 (99.8%)	2 (0.2%)	0 (0%)	0 (0%)
**Requested response**	1188 (96.9%)	38 (3.1%)	0 (0%)	0 (0%)

Supplementary Table 3: *The table delineates row by row which combination of matching variables between the original enquiry dataset and the tumor documentation dataset resulted in how many matches. The last row also indicates the number of distinct matches based on the four combinations.*

**Last name**	**First name**	**Date of Birth**	**Diagnosis**	**Identified Matches**
X	X	X		56
X	X		X	151
X		X	X	44
	X	X	X	43
**Distinct matches**				177
**Identified as false positives**				29

Supplementary Note 1: *Record Linkage Validation*

To assess whether individuals who contacted the central platform subsequently received treatment at one of the two associated university hospitals, a Privacy-Preserving Record Linkage (PPRL) was conducted using standardized identifiers. Initial matching based solely on first name, last name, and date of birth yielded only 56 entries, highlighting frequent inconsistencies or missing values—particularly for birth dates.

To improve match rates, additional combinations of matching variables were used. The most successful combination included last name, first name, and generalized diagnosis (ICD-10 group), which resulted in 151 potential matches. Across all combinations, 177 distinct records were identified as potential matches and subjected to manual validation.

During validation, 29 cases were excluded due to conflicting birth years or ambiguous multiple matches for a single record. When inconsistencies in diagnosis were found between the enquiry dataset and tumor documentation, the diagnosis from the tumor documentation system was considered authoritative and used to correct the entry. Ultimately, 148 validated matches were merged into the analysis dataset and supplemented with additional clinical data (e.g., year of birth, disease entity, treatment date, and partial postal code).

This process improved data completeness and allowed us to analyze follow-up care utilization following initial enquiries.

Supplementary Figure 2: *Percentage of the most frequent tumor localizations. In "unspecific" cases, usually only histology or other information regarding the tumor was provided (e.g. adenocarcinoma), but not the specific tumor type or its location (e.g. colon). Note: 12.2% of diagnoses are unknown (see Supplementary Table 2) and therefore were not included in this graph.*

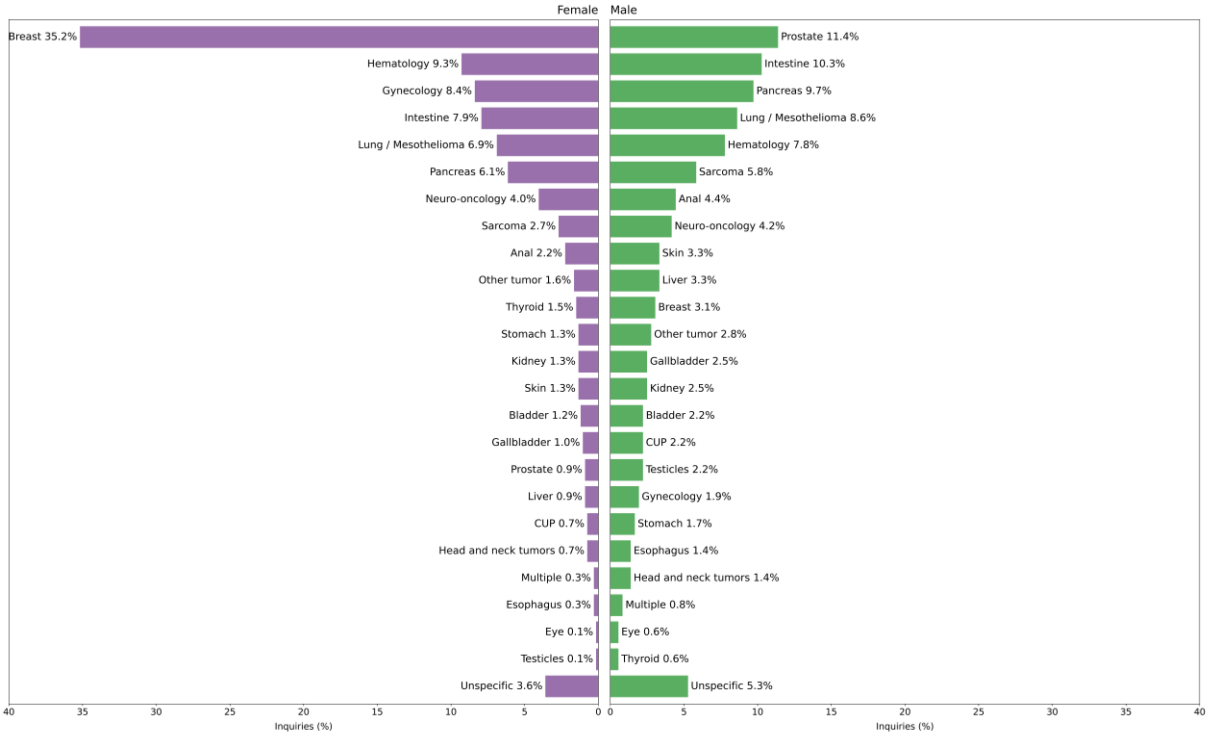

Supplementary Figure 3 left: *Distribution of cancers as reported during the inquiries and / or supplemented by tumor documentation data. In "unspecific" cases, usually only histology or other information regarding the tumor was provided (e.g. adenocarcinoma), but not the specific tumor type or its location (e.g. colon).* ***Supplementary Figure 3 right:*** *Distribution of role within different cancer types*

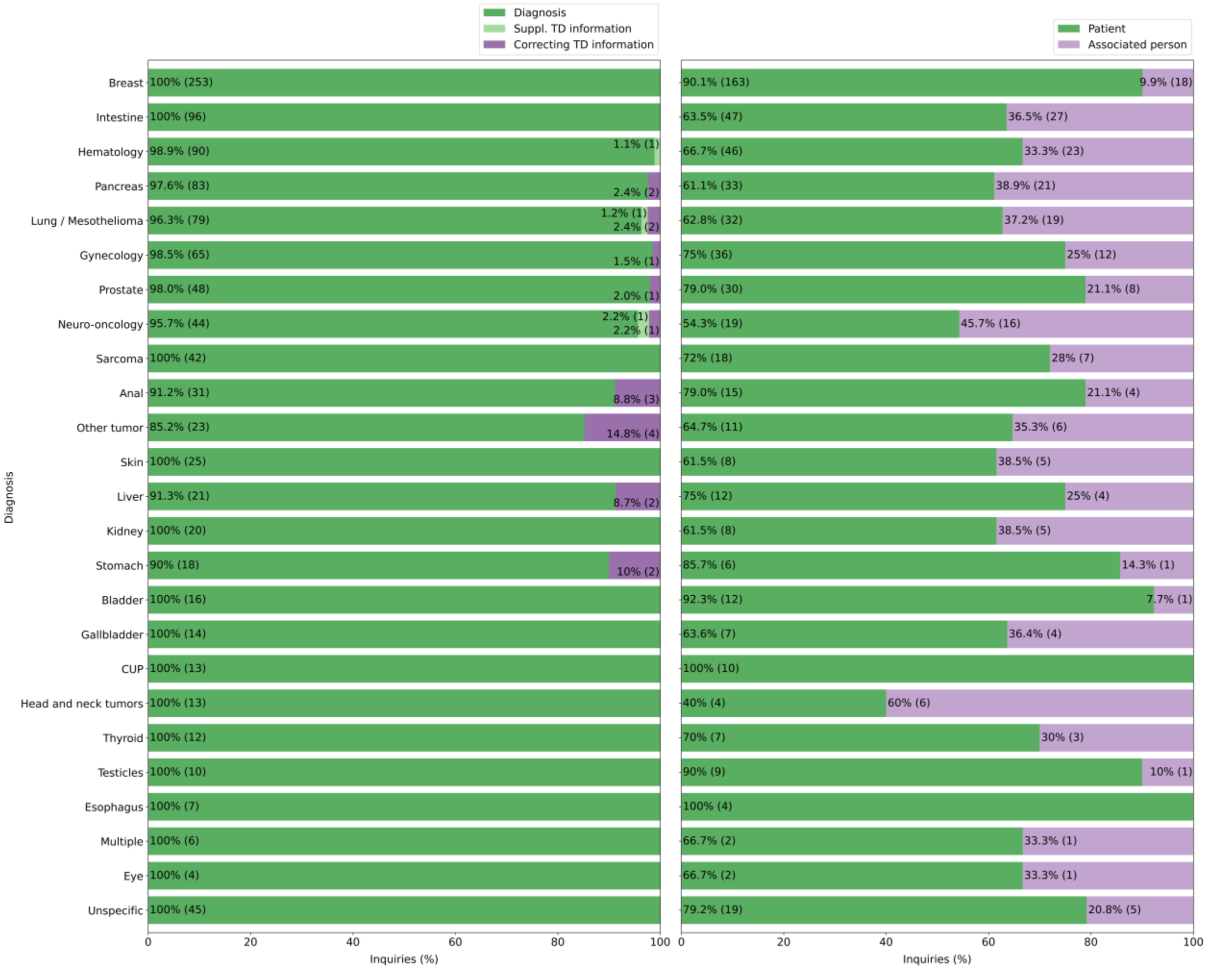

Supplementary Figure 4: *Analysis if a requested supportive care advice was subsequently provided at the CCC supportive care center. (A) Distribution of patients/caregivers with SC consultation and no SC consultation. Distribution of this cohort by age (B) and gender (C).*

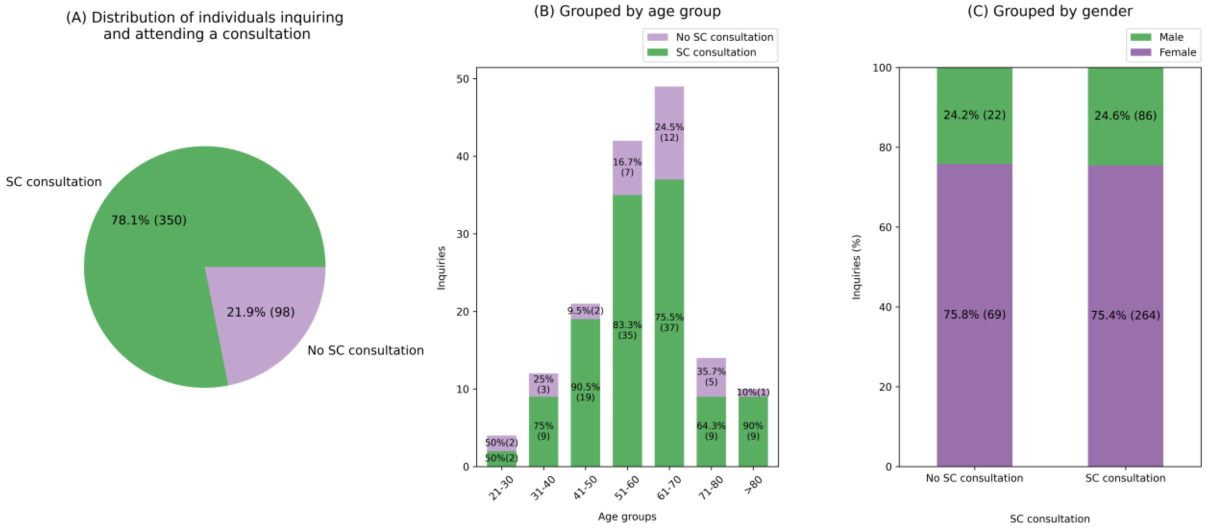

__Supplementary Figure 5:__ Distribution of inquiries based on postcode areas

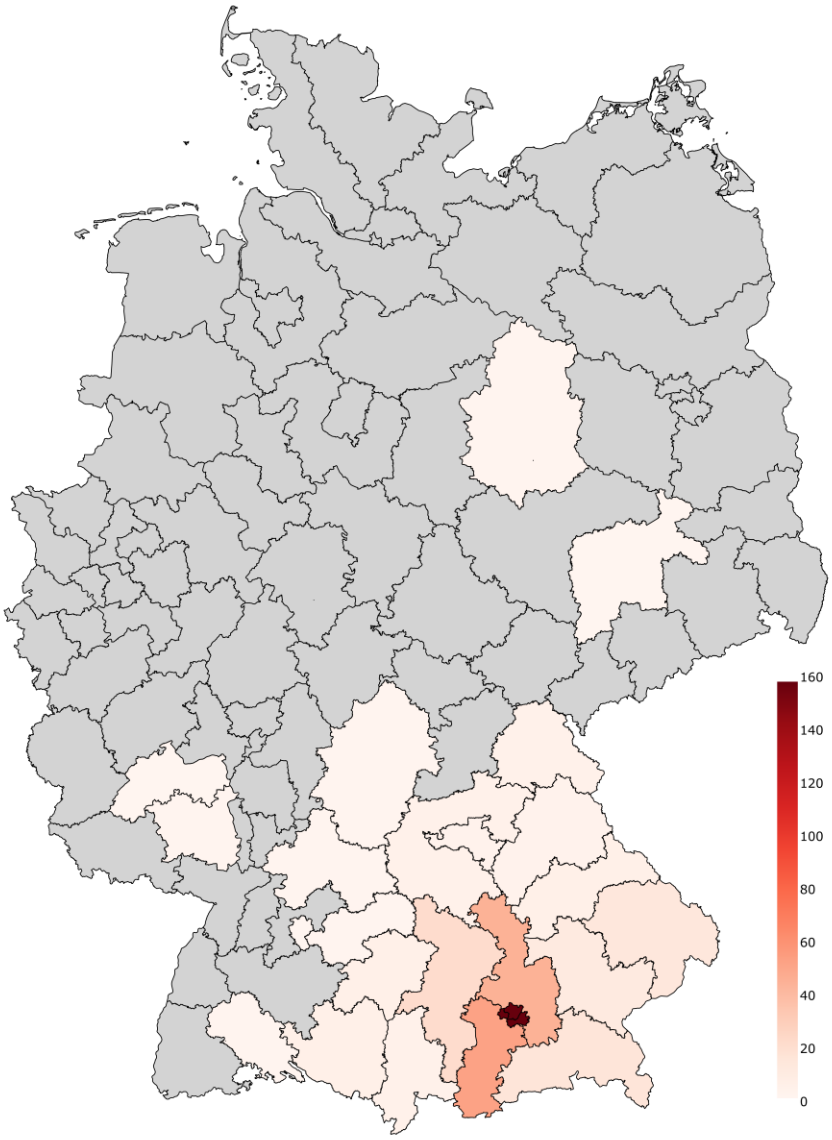

__Supplementary Table 4:__ The table provides information on the geographical origin of the enquiry within Germany.

**Area**	**Total (n = 376)**	**SC consultation (n = 200)**	**Second opinion (n = 122)**	**Treatment (n = 26)**	**Others (n = 21)**	**Study participation (n = 3)**	**Multiple (n = 4)**
**Munich and Munich region**	255 (68%)	157 (79%)	60 (49%)	16 (62%)	17 (81%)	2 (67%)	3 (75%)
**Bavaria (excluding the Munich and Munich region)**	108 (29%)	37 (19%)	56 (46%)	10 (38%)	4 (19%)	0 (0%)	1 (25%)
**Rest of Germany (outside Bavaria)**	13 (3%)	6 (3%)	6 (5%)	0 (0%)	0 (0%)	1 (33%)	0 (0%)
